# Supplementary material for: Influence of electric double layer rigidity on CO adsorption and electroreduction rate
Source: Nat Commun. 2024 Mar 2;15:1926. doi: 10.1038/s41467-024-46318-4 (PMC10908862; doi:10.1038/s41467-024-46318-4)
Supplement: Supplementary file 1 — Supplementary Information [file 41467_2024_46318_MOESM1_ESM.pdf]

Supplementary Information

Influence of Electric Double Layer Rigidity on CO Adsorption and Electroreduction Rate

Jiajie Hou[1], Bingjun Xu[2] & Qi Lu[1]

[1]State Key Laboratory of Chemical Engineering, Department of Chemical Engineering, Tsinghua University, Beijing 100084, China
[2]College of Chemistry and Molecular Engineering, Peking University, Beijing 100871, China

Emails: b_xu@pku.edu.cn; luqicheme@mail.tsinghua.edu.cn

The PDF file includes:

1. Supplementary Figures 1–9
2. Supplementary Table
3. Supplementary Note
4. Supplementary References

Supplementary Figures

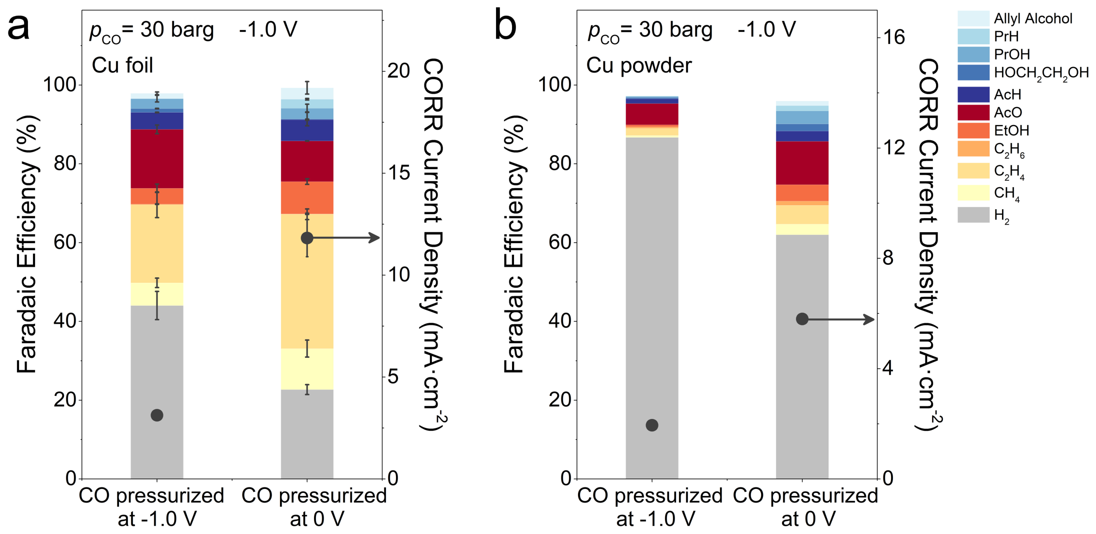

Supplementary Fig. 1. Comparison of CO reduction reactivities at -1.0 V and p_{CO} of 30 barg on (**a**) polycrystalline Cu foil and (**b**) Cu powder electrodes when CO is pressurized at -1.0 V and 0 V in 0.1 M potassium phosphate buffer electrolyte of pH=8. The observed decrease in CORR rate and Faradaic efficiency, in comparison to the Cu foil, is likely due to the exposure of the carbon fiber paper support to the electrolyte, which enhances the competing hydrogen evolution reaction. The error bars represent the standard deviation from at least three independent measurements.

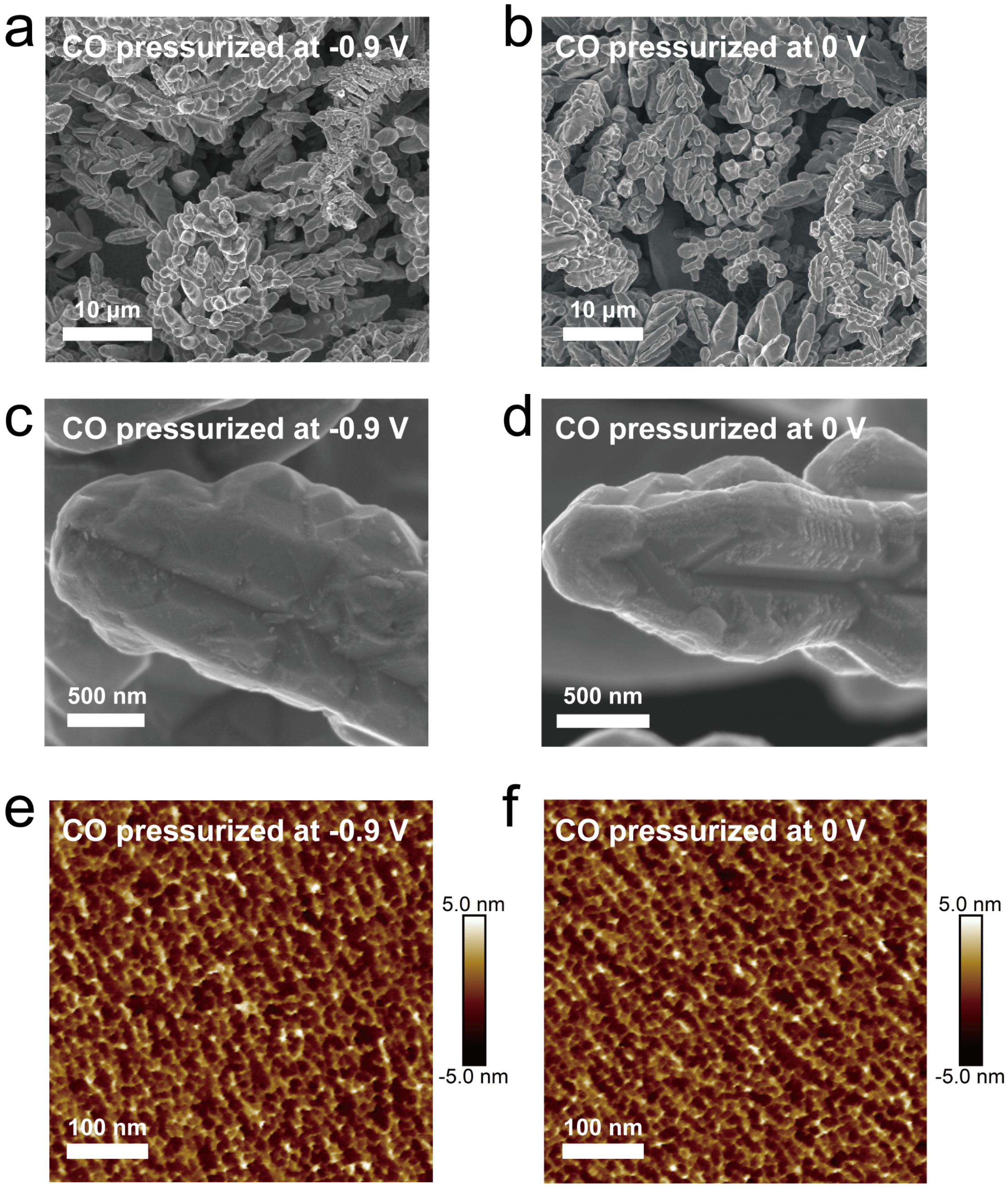

Supplementary Fig. 2. The field-emission scanning electron microscopy images of post-experimental (**a-d**) Cu powder recorded on a Merlin FESEM from Zeiss and AFM height images of post-experimental (**e, f**) Cu foil obtained using Bruker Dimension ICON where (**a, c, e**) CO was pressurized at -0.9 V and (**b, d, f**) CO was pressurized at 0 V.

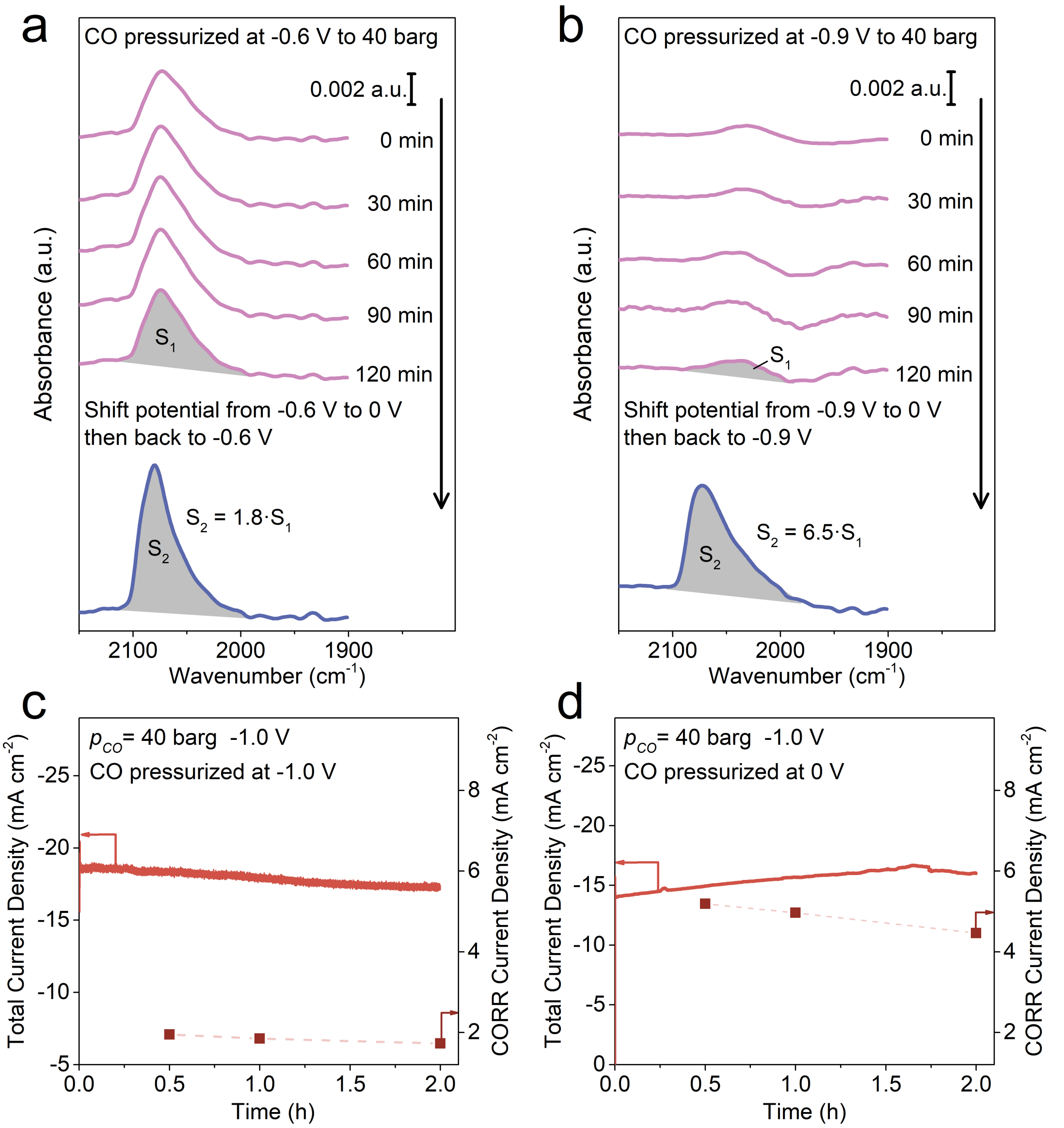

Supplementary Fig. 3. Time dependence of CO_L bands in SEIRAS investigations when CO is pressurized at (**a**) -0.6 V and (**b**) -0.9 V in 0.1 M potassium phosphate buffer electrolyte of pH=8. The CO_L band remained stable throughout at least 2-hour experimental period. Upon shifting the potential to 0 V and then back to -0.6 or -0.9 V, a marked increase in CO_L peak area was observed. Total current density profiles and time dependent CORR Faradaic efficiencies for 2-hour electrolysis on Cu powder electrodes at -1.0 V and 40 barg when CO is pressurized at (**c**) -1.0 V and (**d**) 0 V in 0.1 M potassium phosphate buffer electrolyte of pH=8. These results suggest that EDL_{lp} is stable over the timescale of our experiments (> 2 hours), which is attributable to the significant barrier between EDL_{lp} and EDL_{hp} as depicted in Fig. 2a.

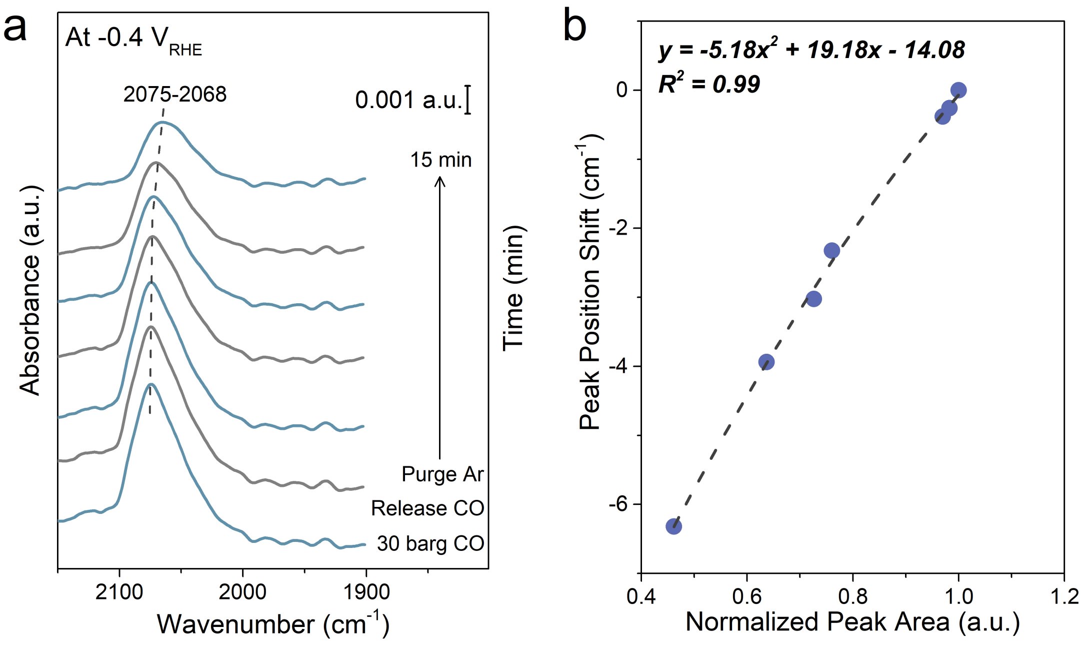

Supplementary Fig. 4. (**a**) In situ SEIRAS investigation of the dependence of CO_L band frequency on peak area, conducted by switching headspace gas from 30 barg CO gas to Ar on polycrystalline Cu at -0.4 V in potassium phosphate buffer of pH 8. (**b**) Peak position shift from that under 30 barg CO, plotted as a function of peak area. The dashed line represents a second-order polynomial fit of the data points. A spectral resolution of 2 cm^{-1} was set for accurate calibration.

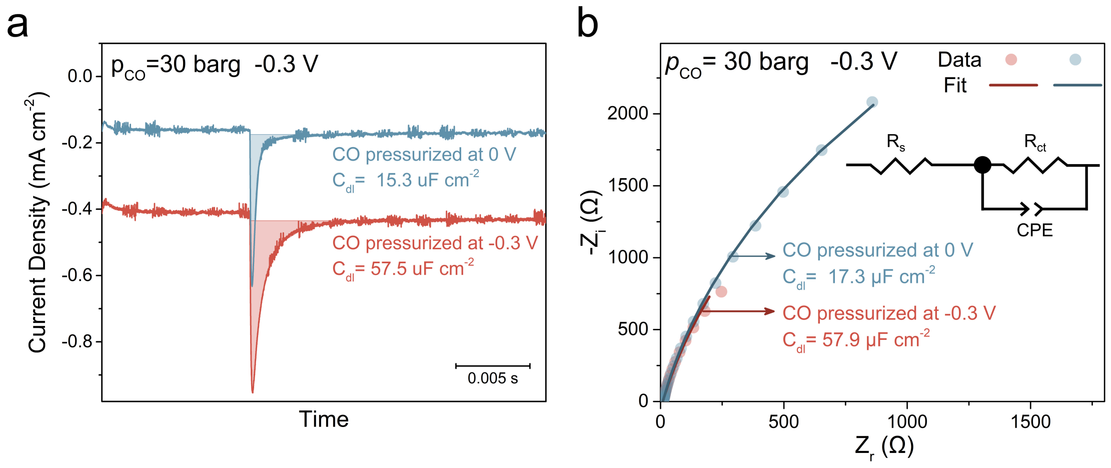

Supplementary Fig. 5. Comparison of specific double layer capacitance at -0.3 V and p_{CO} of 30 barg on polycrystalline Cu foil when CO is pressurized at -0.3 V and 0 V in 0.1 M potassium phosphate buffer electrolyte of pH=8 using (**a**) potential step voltammetry and (**b**) electrochemical impedance spectroscopy.

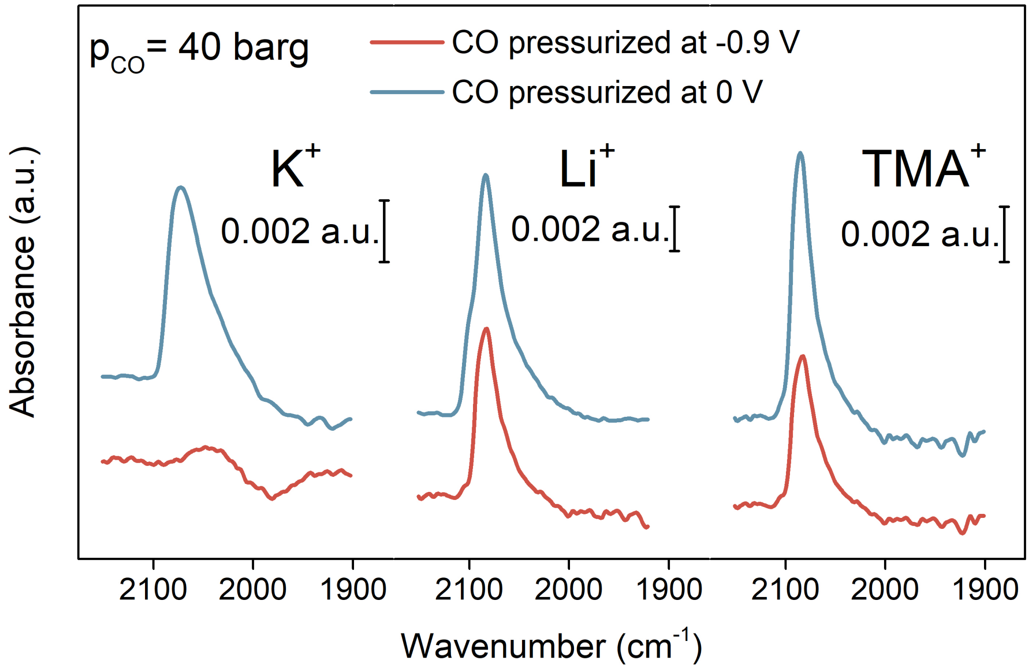

Supplementary Fig. 6. Comparison of ATR-SEIRAS spectra of CO_L band at 40 barg in electrolytes containing K^+, Li^+ and TMA^+, with CO pressurized at -0.9 V (red) and 0 V (blue).

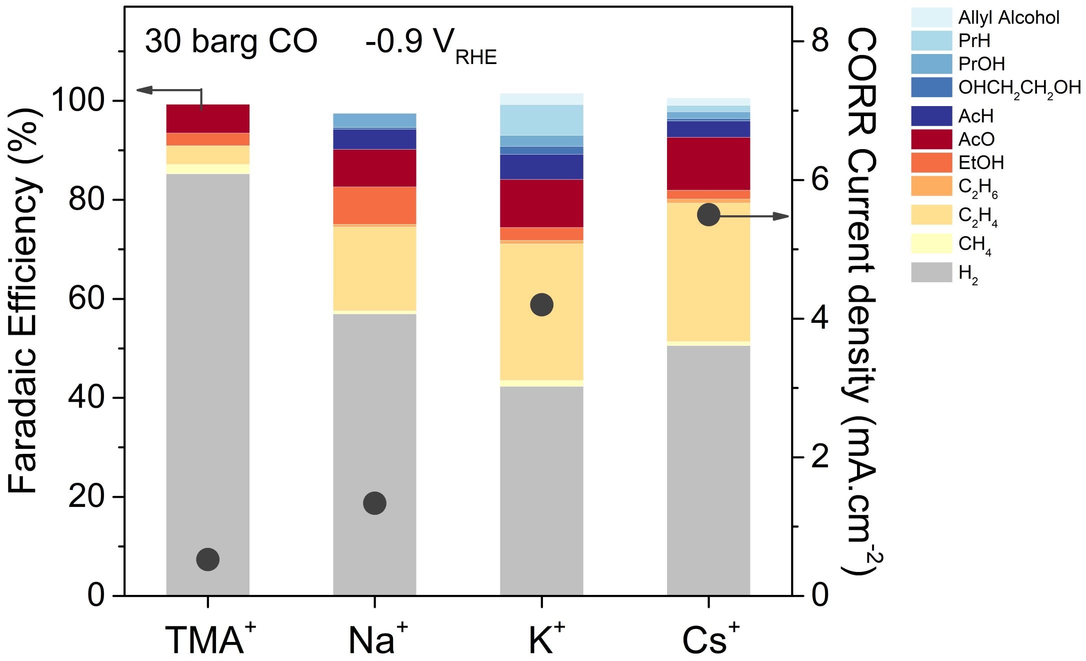

Supplementary Fig. 7. Comparison of CO reduction reactivities at -0.9 V and p_{CO} of 30 barg on polycrystalline Cu foil in the phosphate buffer of pH 8 containing different cations of the same concentration.

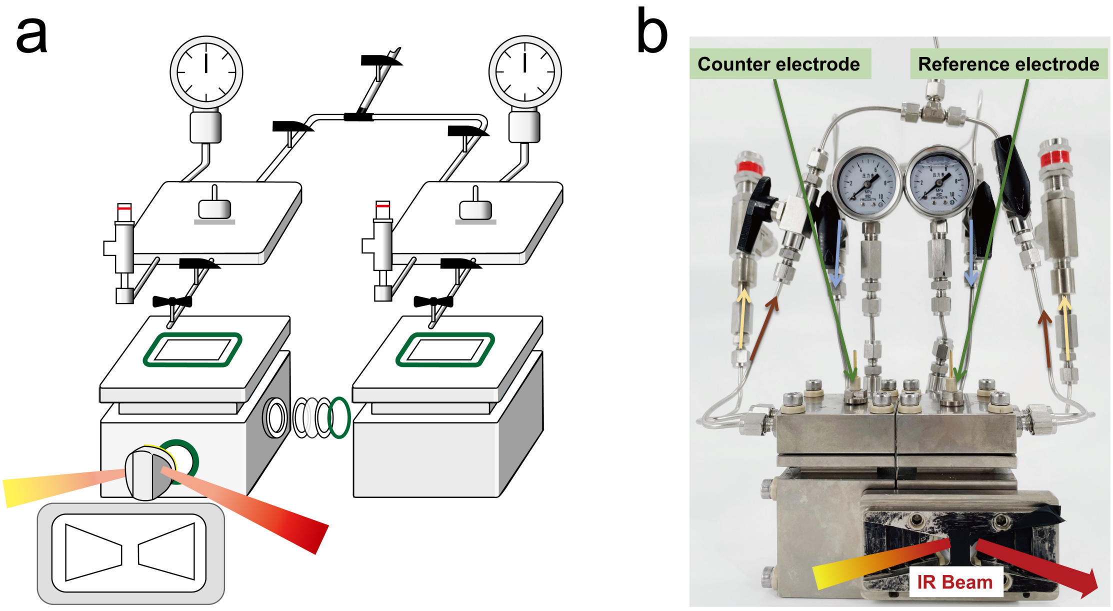

Supplementary Fig. 8. (**a**) Schematic and (**b**) image of the custom-designed high-pressure surface enhanced infrared absorption spectroelectrochemical cell.

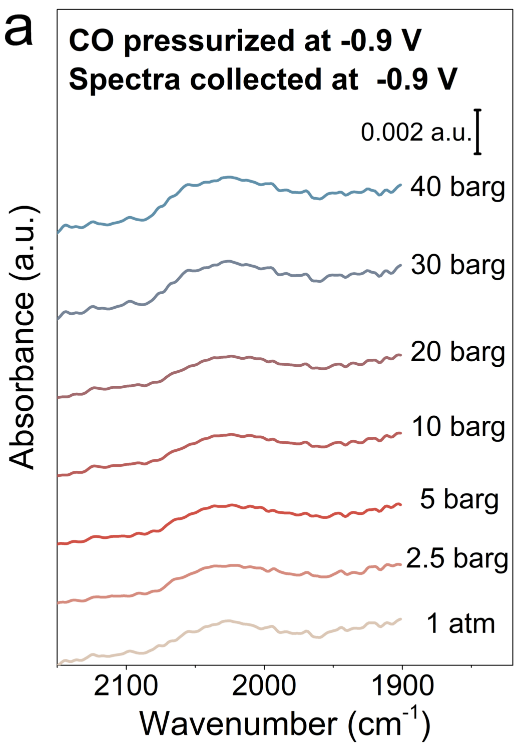

Supplementary Fig. 9. Pressure dependent CO_L band on electrodeposited Cu film electrode at -0.9 V when the CO pressure is elevated at (a) -0.9 V and (b) 0 V in 0.1 M potassium phosphate buffer electrolyte (pH=8).

Supplementary Table

Supplementary Table 1. Simulated impedance parameters of Nyquist plots at -0.3 V in 0.1 M potassium phosphate buffer electrolyte (pH=8) when CO was pressurized at -0.3 V and 0 V. The geometric surface area of Cu foil electrode was 2.2 cm^2.

Condition	R_u /(Ω)	R_{ct} /(Ω)	Y_0 /(μ$\Omega^{-1}\cdot s^n$)	n	C_{eff} /(μF)	C_{EDL} /(μF·cm^{-2})
CO pressurized at -0.3 V	9.3	8875	263.5	0.89	127.4	57.9
CO pressurized at 0 V	9.6	9393	83.3	0.90	38.1	17.3

Supplementary Note

Dipole-coupling correction of CO_L band wavenumbers.

The intrinsic Stark tuning rate was calculated by correcting the shift of wavenumbers caused by dynamic dipole-coupling effect from as-measured wavenumbers. The influence of the dipole-coupling on the CO_L peak position was determined by correlating the relative peak area versus peak position of CO_L at a specified potential, as previously reported.[1] To obtain this correlation, we first collected CO_L band at -0.4 V after CO was pressurized to 30 barg at 0 V and equilibrated for 15 min, obtaining the maximum CO_L peak area in the spectra shown in Fig. 3a. Subsequently, CO was gradually released to reach ambient pressure and then Ar was delivered into the electrolyte to replace the rest of CO. The SEIRA spectra were continuously collected over time, capturing the gradual decrease of the CO_L band and the redshift of the peak position from 2075 to 2068 cm^{-1} due to CO desorption, indicative of reduced dipole-coupling of adsorbed CO (Supplementary Fig. 4a). The correlation between the peak area and peak position was fitted through a second-order polynomial f(x) with a R-squared value of 0.99 (Supplementary Fig. 4b). The wavenumbers of CO_L bands collected at different potentials were then corrected with f(x) according to the following equation:

$$v_{\mathrm{cor.}} = v_0 - f(S/S_{max}) \qquad (1)$$

where $\upsilon_{\mathrm{cor.}}$ and υ_0 are corrected and as-measured wavenumber, respectively. S is the integrated area of CO_L band and S_{max} is the peak area of CO_L band at -0.4 V when CO is pressurized to 30 barg at 0 V. The intrinsic Stark tuning rate could be determined with the corrected wavenumbers ($\upsilon_{\mathrm{cor.}}$).

Supplementary References

1. Chang, X. *et al.* Determining intrinsic stark tuning rates of adsorbed CO on copper surfaces. *Catal. Sci. Technol* **11**, 6825-6831 (2021).
